# Posttranslational modifications of OsRLCK176 as a molecular switch to balance growth and immunity in rice

**DOI:** 10.1093/plcell/koad273

**Published:** 2023-10-26

**Authors:** Leiyun Yang

**Affiliations:** Assistant Features Editor, The Plant Cell, American Society of Plant Biologists; Department of Plant Pathology, College of Plant Protection, Nanjing Agricultural University, Key Laboratory of Integrated Management of Crop Diseases and Pests, Ministry of Education, Nanjing 210095, China; The Key Laboratory of Plant Immunity, Nanjing Agricultural University, Nanjing 210095, China

Growth and immunity are two fundamental biological processes essential for organismal survival. Plants have limited resources, making balancing growth and defense critical. To solve this problem, they have evolved a number of molecular switches to fine-tune responses as needed to balance growth and defense. Critical components of such switches are posttranslational protein modifications such as phosphorylation mediated by protein kinases and ubiquitination mediated by E3 ubiquitin ligases. Upon pathogen infection, receptor-like cytoplasmic kinases (RLCKs) can sense immune signals from plasma membrane–bound immune receptors and phosphorylate downstream target proteins for immune signal transduction, making RLCKs a central hub for immune signaling (DeFalco and Zipfel 2021).

In new work, **Baohui Mou and colleagues (**Mou et al. 2023**)** reveal a mechanism of growth-defense balance through maintaining OsRLCK176 homeostasis via posttranslational modifications by interactions with the calcium-dependent protein kinase OsCPK17. OsCPK17 was previously found to play a crucial role in abiotic stress response (Almadanim et al. 2017). To test whether it is involved in biotic stress response, the authors isolated a T-DNA knockout mutant, *cpk17-1*, and generated an RNA interference line in which *CPK17* transcripts were largely reduced. The disease resistance phenotype was tested in these mutant lines in response to two agronomically important pathogens: bacterial blight pathogen *Xanthomonas oryzae pv. oryzae* (*Xoo*) and bacterial leaf streak pathogen *X. oryzae* pv. *oryzicola* (*Xoc*). These mutants displayed enhanced susceptibility to both pathogens relative to the wild-type (WT) plants, suggesting that OsCPK17 positively regulates rice resistance to pathogenic bacteria.

RLCK VII subfamily member BOTRYTIS-INDUCED KINASE1 (BIK1) is phosphorylated by CPK28 to regulate immunity in Arabidopsis, and OsRLCK57, OsRLCK107, OsRLCK118, and OsRLCK176 are the closest homologs to BIK1 (Wang et al. 2018). The authors hypothesized that OsCPK17 might interact with OsRLCK57, OsRLCK107, OsRLCK118, or OsRLCK176. To test this, the authors performed a split luciferase complementation analysis and found only OsRLCK176 interacted with OsCPK17. Immunoblot assays revealed that OsRLCK176 abundance was significantly decreased in the *cpk17* mutant, indicating OsCPK17 directly interacts with and stabilizes OsRLCK176. These findings prompted the authors to test whether OsRLCK176 is a substrate of OsCPK17. In vitro kinase assays and immunoblot assays revealed that OsRLCK176 was indeed phosphorylated by OsCPK17, and this phosphorylation was reduced in the *cpk17* mutant. They further identified the phosphorylated residue Ser83 by liquid chromatography-tandem mass spectrometry. To test whether Ser83 phosphorylation is associated with OsRLCK176 accumulation, they generated OsRLCK176^S83D^, a phospho-mimetic mutant of OsRLCK176, and found OsRLCK176^S83D^ was more abundant than WT OsRLCK176 in the *cpk17* mutant background. Using a site-specific antibody against OsRLCK176^pSer83^, they detected Ser83 phosphorylation in OsRLCK176 only in WT but not in the *cpk17* mutant. Collectively, these data support that phosphorylation of OsRLCK176 at Ser83 by OsCPK17 contributes to OsRLCK176 stability.

Next, they tested whether Ser83 phosphorylation of OsRLCK176 is involved in plant immunity by analyzing the immunity phenotypes of transgenic plants expressing phospho-mimetic OsRLCK176^S83D^ and WT OsRLCK176. After *Xoo* and *Xoc* infection, OsRLCK176^S83D^-expressing transgenic plants exhibited elevated resistance and higher induced defense gene expression compared to OsRLCK176-expressing transgenic plants. Additionally, OsRLCK176^S83D^ conferred the *cpk17* mutant an enhanced resistance to *Xoo* and *Xoc*. These findings suggested that phosphorylation of OsRLCK176 at Ser83 by OsCPK17 promotes its immune function in rice.

Because the stability of OsRLCK176 is regulated in vivo and plant U-box (PUB) E3 ligases are important components in protein degradation system, the authors performed a yeast two-hybrid screen using a rice *PUB* gene mini-library to identify putative PUB proteins responsible for OsRLCK176 degradation, and OsPUB12 was identified as a positive interactor. Immunoblot assays showed that OsRLCK176 degraded much quicker when it was coexpressed with OsPUB12, and the endogenous level of OsRLCK176 was significantly higher in the *pub12* mutant. Further analyses showed that OsRLCK176 was ubiquitinated by OsPUB12 for degradation. Importantly, ubiquitination was inhibited by the S83D mutation in OsRLCK176. As expected, the *pub12* mutants had higher defense marker gene expression and reactive oxygen species burst than WT, as well as elevated resistance to *Xoo* and *Xoc*.

Based on these findings, the authors proposed a model where, under normal conditions, OsPUB12 degrades OsRLCK176 and represses defense responses. Upon defense activation, OsCPK17 phosphorylates and stabilizes OsRLCK176 for immune activation (see Figure 1). This work showcases a new mechanism of growth-defense balance and advances our understanding of posttranslational modifications in growth-defense balance.

**Figure 1.**
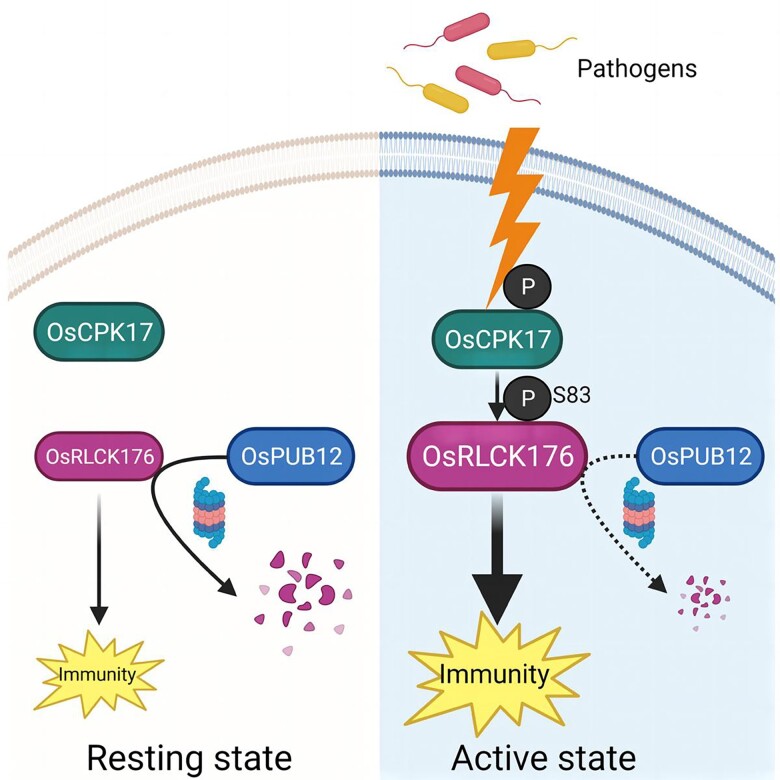
Proposed model for OsRLCK176-mediated growth-defense balance in rice. In the absence of pathogen infection (resting state), OsRLCK176 is ubiquitinated by OsPUB12 and degraded, and rice immunity is maintained at a low level. Upon defense activation, OsCPK17 is activated and phosphorylates OsRLCK176 at Ser83, which inhibits OsRLCK176 ubiquitination, resulting in OsRLCK176 accumulation and enhanced defense activation. Reprinted from Mou et al. (2023) Figure 8.

## Dive Curated Terms

The following phenotypic, genotypic, and functional terms are of significance to the work described in this paper:

CPK28 Gramene: AT5G66210
CPK28 Araport: AT5G66210

CPK17 Gramene: AT5G12180
CPK17 Araport: AT5G12180

PUB12 Gramene: At2g28830
PUB12 Araport: At2g28830
